# Outcome of hospitalized patients with COVID-19 and predictors at the Buea Regional Hospital, South West region of Cameroon

**DOI:** 10.11604/pamj.2024.47.165.34572

**Published:** 2024-04-04

**Authors:** Clovis Nkoke, Cyrille Nkouonlack, Denis Teuwafeu, Ronald Gobina, Ahmadou Musa Jingi, Jerry Aseneh, Susan Enyoue, Laura Folefac, Divine Martin Mokake, Vincent Verla

**Affiliations:** 1 Faculty of Health Sciences, University of Buea, Buea, Cameroon,; 2 Buea Regional Hospital, Buea, Cameroon,; 3 Faculty of Health Sciences, University of Bamenda, Bamenda, Cameroon

**Keywords:** COVID-19, co-morbidities, mortality, Cameroon

## Abstract

**Introduction:**

there is a paucity of data on the epidemiology of COVID-19 infection in Cameroon with a few studies limited to big urban cities. The objective of this study was to describe the clinical characteristics and outcomes of hospitalized patients with COVID-19 at the Buea Regional Hospital, in the South West region of Cameroon.

**Methods:**

this was a retrospective cross-sectional study. The medical records of hospitalized patients with COVID-19 were reviewed from 2020 to 2021. Hospitalized patients with laboratory-confirmed COVID-19 were included. Binary logistic regression was used to identify factors associated with mortality.

**Results:**

two hundred and ten (210) patients were included in this cohort. There were 114 (54.7%) men. The mean age was 60±17.1 years. The common co-morbidities were hypertension (46.7%), diabetes mellitus (31%), and HIV infection (5.7%). The most common symptoms were dyspnea (93.3%), fatigue (93.8%), cough (77.6%), and fever (71.4%). The median oxygen saturation was 85% and the median respiratory rate was 24 cycles per minute. More than 80% had crackles on lung examination. Death occurred in 57 (27.1%) patients. In binary logistic regression, the factors independently associated with mortality were heart failure (aOR: 6.7, p=0.034), SBP < 100 mmHg (aOR: 8.1, p<0.001), RR > 24 cpm (aOR: 3, p=0.016), SaO_2_<90% (aOR: 6.2, p=0.031), blood glucose > 150mg/dL (aOR: 3.3, p=0.02), and CRP > 50 mg/L (aOR: 3.3, p=0.036). For every 1 mg/dL rise in blood glucose, the odds of death increased by 1% (p=0.011). For every 1 mg/L rise in the C-reactive protein (CRP), the odds of death increased by 1% (p=0.054).

**Conclusion:**

over half of hospitalized patients with laboratory-confirmed COVID-19 infection in the South West Region of Cameroon were males. Hypertension and diabetes were common co-morbidities. More than a quarter of these patients died. Furthermore, having heart failure, low systolic blood pressure (SBP), low oxygen saturation, elevated respiratory rate, high CRP and blood glucose levels on admission were associated with poor prognosis.

## Introduction

Coronavirus disease 2019 (COVID-19) caused by a novel coronavirus named the Severe Acute Respiratory Syndrome Coronavirus 2 (SARS-CoV-2) was initially reported in Wuhan in December 2019, China, and rapidly became a global pandemic [1]. As of December 21^st^ 2021, there have been approximately 274,628,461 confirmed cases of COVID-19 globally, including 5,358,978 deaths [2]. The clinical manifestations may vary from mild to severe symptoms in COVID-19 patients. Patients with multiple co-morbidities are at increased risk of poor outcomes [3]. The first patient with confirmed COVID-19 in Cameroon was reported on the 6^th^ of March 2020 [4]. Shortly afterward, the infection rapidly spread throughout the country including the South West region of Cameroon, which is in very close proximity to Douala, the economic capital of Cameroon and the main gateway into the country.

Specialized COVID-19 treatment centers and laboratories were set up for the diagnosis and management of COVID-19 in Cameroon. The Buea Regional Hospital in the South West region of Cameroon harbored the main treatment center in the region and received cases from other districts in the region. Reports on the epidemiology of hospitalized patients with COVID-19 in Cameroon are scarce. Buea was an early epicenter of the COVID-19 pandemic in Cameroon, yet data on hospitalized patients with COVID-19 is lacking. To further our understanding of the disease, it is important to ascertain whether the epidemiological features of COVID-19 in Africa differ from those in other regions of the world. This study aimed to examine the clinical characteristics and outcomes of hospitalized patients with COVID-19 at the Buea Regional Hospital.

## Methods

**Study design and setting:** this was a retrospective cross-sectional study conducted at the COVID-19 treatment center of the Buea Regional Hospital. This is a secondary-level hospital that serves as one of the two main referral hospitals in the South West region of Cameroon and the main teaching hospital of the Faculty of Health Sciences of the University of Buea. The hospital does not have an intensive care unit capacity and there are no ventilators. There is no chest physician. The PCR tests for COVID-19 were performed in the laboratory of the University of Buea which received an accreditation from the Cameroon Ministry of Public Health. All the COVID-19 Tests were performed with a nasopharyngeal specimen and obtained by a trained laboratory technician. All the other laboratory investigations were performed in the Buea Regional Hospital laboratory. The COVID-19 treatment and isolation unit had a capacity of 21 beds. It serves a catchment population of about 300,000 inhabitants. Buea is a semi-urban setting and the main economic activity is agriculture.

**Study population:** we included hospitalized patients with COVID-19 from March 2020 to November 2021 diagnosed with a positive PCR test. The patients admitted to the unit were those with moderate and severe disease.

**Data collection:** we collected data on socio-demographic characteristics, co-morbidities and clinical presentation, laboratory, and outcome parameters. Socio-demographic variables collected included gender and age. The laboratory data included routine blood tests, such as complete blood count, biochemistry tests (serum creatinine, glycemia) and infection-related parameters (C reactive protein) that were assessed at the time of admission.

**Ethical consideration:** approval was obtained from the institutional board review of the Buea Regional Hospital, IBRN: MPHSWRDPH/BRH/IRB/202. No informed consent was required. The need for consent was waived since it was a retrospective study from the medical records. All data were fully anonymized.

**Data analysis:** we analyzed the data using IBM SPSS version 26 (IBM Corp, Armonk, NY, USA). Continuous variables are presented as means with standard deviation (SD) or median with inter-quartile range (IQR), and discrete variables as frequencies and proportions. We compared means with independent sample T-test and proportions with Chi-squared or Fischer exact test where appropriate. Factors associated with poor outcomes (death) were assessed in bivariate analyses. Factors independently associated with poor outcomes were assessed using binary logistic regression. We used three different models for the co-morbidities, the parameters on admission, and the biochemical parameters on admission. Factors with a p-value <0.2 were entered into the model. For the co-morbidities, factors were hypertension, diabetes, heart failure, alcohol consumption, and Chronic obstructive pulmonary disease (COPD). For the parameters on admission, the variables entered were systolic BP < 100 mmHg (shock threshold), diastolic BP < 60 mmHg (shock threshold), heart rate > 90 bpm (tachycardia threshold), respiratory rate > 24 cpm (median RR), capillary saturation (SaO_2_) < 90% (critical threshold), and fever. For the biochemical parameters, the variables entered were: blood glucose > 150 mg/dL (significant hyperglycemia), Hb< 12 g/dL (anemia), WBC > (10000/mm3), serum creatinine > 15 mg/L (significant renal impairment), and CRP > 50 mg/L (severe inflammation). A p-value less than 0.05 was considered significant.

## Results

**Figure 1:**
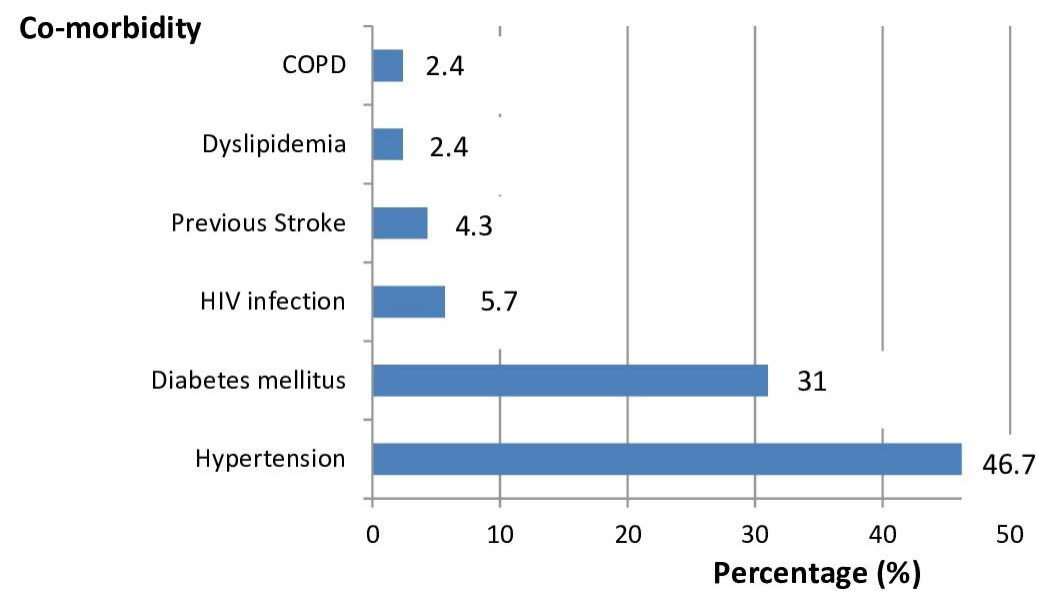
distribution of co-morbidities

**Socio-demographic characteristics and co-morbidities:** two hundred and ten (210) patients were included in the study, of which 54.7% were men and the mean age was 60±17.1 years, Table 1. Among the women admitted, one was pregnant. The mean age was 60±17.1 years. There was no significant age difference between men and women (61 years vs 58.7 years, p=0.34). The most commonly reported co-morbidities (Figure 1) were hypertension (46.7%), diabetes mellitus (31%), HIV infection (5.7%), and previous stroke (4.3%).

**Table 1:** socio-demographic characteristics and co-morbidities

Variables	Frequency (n)	Percentage (%)
Age, mean (SD), years	60 (17.1)	NA
Sex, male	114	54.7
**Co-morbidities**		
Hypertension	98	46.7
Diabetes mellitus	65	31
HIV infection	12	5.7
Stroke	9	4.3
Heart failure	6	2.9
Alcohol	5	2.4
Dyslipidemia	5	2.4
COPD	5	2.4
Smoking	4	1.9
Atrial fibrillation	3	1.4
Ischemic heart disease	3	1.4
Chronic kidney disease	3	1.4

**Clinical characteristics, laboratory parameters, and outcome:** the clinical characteristics on admission are shown in Table 2. The most common symptoms were dyspnea (93.3%), fatigue (93.8%), cough (77.6%), and fever (71.4%). The mean systolic BP was 130mmHg and the mean diastolic BP was 81mmHg. The median oxygen saturation was 85% and the median respiratory rate was 24 cycles per minute. More than 80% had crackles on lung examination. The biochemical parameters on admission are shown in Table 3. There was a marked increase in blood glucose and CRP on admission.

**Table 2:** clinical characteristics on admission and outcome

Variables	Frequency (n), mean (SD)	Percentage (%)
**Parameters on admission**		
SBP, mean±SD (mmHg)	130.1 ± 25.2	NA
DBP, mean ±SD (mmHg)	81.3 ± 15.9	NA
Heart rate (bpm), median (IQR)	92 (82–103)	NA
Respiratory rate (cycles/min) median (IQR)	24 (20–28)	NA
SaO2(%), median (IQR)	85 (75–93)	NA
**Symptoms and signs on admission**		
Fatigue	197	93.8
Dyspnea	196	93.3
Crackles	182	86.7
Cough	163	77.6
Fever	150	71.4
Headache	20	9.5
Diarrhea	11	5.2
Oedema	7	3.3
Seizure	3	1.4
**Outcome**		
Length of stay (days), median (IQR)	6(4–10)	
Death	57	27.1

**Table 3:** laboratory parameters on admission

Variables	Mean ± SD	Median (IQR)
Hemoglobin (g/dL)	12.3 ± 2.3	12.4 (11.1 - 13.8)
WBC (counts/mm^3^)	8649.7 ± 52229.3	7400 (5400 - 10800)
Platelet (counts/mm^3^)	212555.5 ± 90843.7	196000 (149 - 271000)
Serum creatinine (mg/dL)	15.2 ± 15.2	11 (9 - 15)
CRP (mg/L)	100.5 ± 93.6	76.5 (24 - 192)
Glycemia (mg/L)	246.3 ± 137.3	221 (135 - 299)

WBC = white blood cells, CRP = C-reactive protein

**Factors associated with poor outcome:** the poor outcome variable (death) occurred in 57 (27.1%) patients admitted with COVID-19. The blood glucose was significantly higher in those with poor outcomes (297.2 ± 154.5 Vs 213.4 ± 114.9, p=0.013). The CRP was also significantly higher in those with poor outcomes (131 ± 100.6 Vs 83 ± 85.8, p=0.024). There was no significant difference in serum creatinine and FBC parameters between those with poor and good outcomes (all p-values >0.05). In bivariate analyses, the factors associated with poor outcome (Table 4, Table 4.1 and Table 4.2) were heart failure (OR: 5.8, p=0.025), systolic BP (SBP) < 100 mmHg (OR: 7.9, p<0.001), diastolic BP (DBP)< 60 mmHg (OR: 5.7, p<0.001), heart rate > 90 bpm (OR: 2.1, p=0.028), respiratory rate (RR)> 24 cpm (OR: 7.6, p<0.001), capillary SaO_2_< 90% (OR: 6.9, p<0.001), admission blood glucose > 150 mg/dL (OR: 4.1, p=0.017), anemia (OR: 2.5, p=0.039), and CRP > 50mg/L (OR: 3.6, p=0.018). In binary logistic regression (Table 5), the factors independently associated with poor outcome were heart failure (aOR: 6.7, p=0.034), SBP < 100 mmHg (aOR: 8.1, p<0.001), RR > 24 cpm (aOR: 3, p=0.016), SaO_2_<90% (aOR: 6.2, p=0.031), blood glucose > 150mg/dL (aOR: 3.3, p=0.02), and CRP > 50 mg/L (aOR: 3.3, p0.036). For every 1 mg/dL rise in blood glucose, the odds of death increased by 1% (p=0.011). For every 1 mg/L rise in the CRP, the odds of death increased by 1% (p=0.054).

**Table 4:** factors associated with poor outcome (death) in bivariate analyses

Variables	Frequency (%)	OR (95%CI)	p-value
**Socio-demography**			
**Age ≥ 60 years**			
Yes	36 (29.5)	1.4 (0.8-2.6)	0.3
No	20 (23)	1	
**Sex**			
Male	30 (26.6)	0.97 (0.5-1.8)	0.9
Female	26 (27.1)	1	
**Co-morbidities**			
**Hypertension**			
Yes	32 (32.7)	1.8 (1-3.3)	0.07
No	24 (21.6)	1	
**Diabetes**			
Yes	23 (35.4)	1.8 (1-3.5)	0.06
No	33 (22.9)	1	
**HIV**			
Yes	3 (25)	0.9 (0.2-3.5)	0.9
No	53 (26.9)	1	
**Stroke**			
Yes	2 (22.2)	0.8 (0.2-3.8)	0.8
No	54 (27)	1	
**Heart failure**			
Yes	4 (66.7)	5.8 (1.03-32.6)	0.025
No	52 (25.6)	1	

**Table 4.1:** factors associated with poor outcome (death) in bivariate analyses

Alcohol			
Yes	3 (60)	4.3 (0.7-26.3)	0.09
No	53 (26)	1	
**Dyslipidemia**			
Yes	2 (40)	1.9 (0.3-11.4)	0.5
No	54 (26.3)	1	
**COPD**			
Yes	3 (60)	4.3 (0.7-26.3)	0.09
No	53 (26)	1	
**Smoking**			
Yes	2 (50)	2.8 (0.4-20.3)	0.29
No	54 (26.3)	1	
**Atrial fibrillation**			
Yes	1 (33.3)	1.4 (0.1-15.4)	0.8
No	55 (26.7)	1	
**Ischemic heart disease**			
Yes	1 (33.3)	1.4 (0.1-15.4)	0.8
No	55 (26.7)	1	
**Admission parameters**			
**Systolic BP<100 mmHg**			
Yes	17 (68)	7.9 (3.2-19.7)	<0.001
No	39 (21.2)	1	
**Diastolic BP <90 mmHg**			
Yes	9 (64.3)	5.7 (1.8-17.7)	0.001
No	47 (24.1)	1	
**Heart rate > 90 bpm**			
Yes	39 (33.1)	2.1 (1.1-4)	0.028
No	17 (19.3)	1	

**Table 4.2:** factors associated with poor outcome (death) in bivariate analyses

SaO2 < 90%			
Yes	49 (38.6)	6.9 (2.6 -18.5)	<0.001
No	5 (8.3)	1	
**RR > 24 cpm**			
Yes	35 (50)	7.6 (3.3 -17.5)	<0.001
No	9 (11.7)	1	
**Fever**			
Yes	44 (29.3)	1.6 (0.8 -3.4)	0.186
No	12 (20.3)	1	
**Biochemical parameters**			
**Glycaemia > 150mg/dL**			
Yes	27 (47.4)	4.1 (1.2 -13.5)	0.017
No	4 (18.2)	1	
**Hemoglobin < 12 g/dL**			
Yes	16 (41)	2.5 (1.04 -6.1)	0.039
No	13 (21.7)	1	
**WBC > 10000 cell/mm^3^**			
Yes	11 (37.9)	1.8 (0.7 -4.6)	0.194
No	18 (25)	1	
**Platelets < 150000/mm^3^**			
Yes	10 (38.5)	1.8 (0.7 -4.8)	0.202
No	19 (25.3)	1	
**Creatinine > 15 mg/dL**			
Yes	10 (43.5)	2.1 (0.8 -5.6)	0.138
No	18 (26.9)	1	

CRP = C-reactive protein, BP = Blood pressure, SaO2 = oxygen saturation, RR = respiratory rate, COPD = Chronic Obstructive Pulmonary Disease

**Table 5:** factors independently associated with poor outcome (death) in binary logistic regression

Variable	aOR (95% Confidence interval)	p-value
Hypertension	1.4 (0.7-2.8)	0.294
Diabetes	1.7 (0.8-5.4)	0.146
Heart failure	6.7 (1.6-38.9)	0.034
Alcohol consumption	4.9 (0.8-31)	0.092
COPD	4.6 (0.7-29.1)	0.108
Systolic BP < 100 mmHg	8.1 (2.4-27.1)	0.001
Diastolic BP < 60 bpm	1.5 (0.3-8.6)	0.642
RR > 24 cpm	3 (1.2-7.2)	0.016
SaO_2_< 90%	6.2 (1.2-32.7)	0.031
Fever	1.4 (0.6-3.3)	0.44
Admission glycemia> 150 mg/dL	3.3 (1.6-6.9)	0.02
Serum creatinine > 15 mg/L	2.8 (0.9-9)	0.09
CRP > 50 mg/L	3.3 (1.1-9.9)	0.036

* aOR = adjusted Odds Ratio, CRP = C-reactive protein, BP = Blood pressure, SaO2 = oxygen saturation, RR = respiratory rate, COPD = Chronic Obstructive Pulmonary Disease

## Discussion

This is the first report on the epidemiological characteristics of hospitalized COVID-19 patients in the South West region of Cameroon. Our results show that there was a slight male predominance. The main co-morbidities were hypertension and diabetes. About a quarter of hospitalized COVID-19 patients died. Independent predictors of mortality were, heart failure, SBP < 100 mmHg RR > 24 cpm, SaO_2_<90%, blood glucose > 150mg/dL and CRP > 50 mg/L

The male predominance in those hospitalized with COVID-19 in our setting was similar to that reported in previous studies in two metropolitan cities in Cameroon shortly after the pandemic was reported in the country [5,6]. The mean age of our cohort was 60 years. The patients in this study were older than those reported by Mekolo *et al*. in Douala who had a mean age of 51 years [5]. Gebremariam *et al*. in Ethiopia also reported a younger population with a mean age of 50 years [7]. Global trends have demonstrated differences in demographic characteristics including sex (higher among males) and age (increased risk of severe disease in individuals with advanced age) among COVID-19 patients [8-12]. COVID-19 patients in this cohort were younger compared to some studies in Europe [8,11,12]. Several studies from different countries have demonstrated an age-associated vulnerability to COVID-19 infection in elderly individuals [13].

Hypertension was the most common co-morbidity in our study, reported in 46.7% of the participants. This was considerably higher than the 18% reported in another study in the capital city of Cameroon [6]. It was however lower than that reported in another cohort in the economic capital of Cameroon where the prevalence of hypertension was 68.5% [5]. We also reported a significantly higher prevalence of diabetes (31%) compared to Fouda Mbarga *et al*. who reported a prevalence of diabetes of 5.8% in Yaounde [6]. The prevalence of diabetes in our study was however comparable to that reported by Mekolo *et al*. (37%) in Douala [5]. Hypertension and diabetes were also among the commonest co-morbidities reported in other African countries [14-16]. Globally, hypertension and diabetes have been demonstrated to be among the commonest co-morbidities in patients hospitalized with COVID-19 [8-11]. In China, the most common co-morbidity in hospitalized patients with COVID-19 was diabetes [1,3]. In contrast, hypertension was the most common co-morbidity in America [9,6]. Hypertension and diabetes are significant risk factors for COVID-19 infection and these patients are at increased risk of developing severe forms of the disease [10].

The most common symptoms in our cohort were cough fatigue, cough, and fever. This symptom profile in our cohort was largely consistent with previous studies in Cameroon and other parts of the world [5,8,17]. Regarding laboratory parameters, the mean CRP on admission was significantly elevated as well as the glycemia. This was in accordance with other studies that have reported an elevation in CRP, D-dimers, and other laboratory markers of inflammation [10,11]. The markers of inflammation are shown to be higher in patients with severe disease [12].

The case fatality in hospitalized COVID-19 patients in our study was 27%. This was comparable to the case fatality rate of 32% reported in the economic capital of Cameroon [5]. However, this contrasts with the case fatality rate reported by another study from Cameroon where the authors reported a low case fatality rate of 1% [6]. In that study, only 15% of the patients had a severe form of the disease which can partly explain the low case fatality rate of 1% [6]. The independent predictors of mortality in our cohort were heart failure, low systolic blood pressure, respiratory rate, low oxygen saturation, elevated blood sugar, and elevated CRP. The case fatality rate in our study was comparable to that reported in an Italian study with the authors reporting a 26% mortality rate [18]. Previous studies have demonstrated that age, male sex, and co-morbidities including cardiovascular disease are associated with poorer COVID-19 hospitalization outcomes [10,16,19]. Data from the United Kingdom demonstrated that advanced age was the strongest risk factor for death and outweighed any other demographic characteristics or co-morbidity [20]. In one study from Cameroon, age and male gender were significant predictors of mortality [6]. In our study, neither age nor sex were significant predictors of mortality. Reports from Asia, Europe, and America have presented evidence for an increased risk of mortality with increasing age. This includes higher case fatality rates and deaths per 100,000 individuals in aging populations [13]. The co-morbidity that was significantly associated with mortality in our study was heart failure. Studies have shown that in hospitalized patients with COVID-19, heart failure is an independent predictor of in-hospital mortality [21]. Several laboratory values are prognostic markers in patients with COVID-19. D-dimer > 2•5 µg/mL or levels of CPR above 200 mg/L are strongly associated with mortality [22]. In our cohort, elevated CRP> 50 mg/L and elevated glycemia were independent laboratory predictors of mortality. This finding was similar to results from a Spanish cohort where CRP was a predictor of mortality [11].

**Limitation:** our study has some limitations. The disease severity was not classified according to WHO criteria. Secondly, the relatively small sample size may fail to identify significant associations between the variables. Despite these limitations, our study is the first in the region on this new disease and adds to the existing body of knowledge on COVD-19 epidemiology.

## Conclusion

Over half of hospitalized patients with laboratory-confirmed COVID-19 infection in the South West region of Cameroon were males. Hypertension and diabetes were common co-morbidities. More than a quarter of these patients died. Furthermore, having heart failure, low SBP, low oxygen saturation, elevated respiratory rate, high CRP and blood glucose levels on admission were associated with poor prognosis.

### What is known about this topic

- *COVID-19 is global public health emergency;*
- *Africa seems to be less affected;*
- *Patients with co-morbidities are at high risk of severe infection and poor outcome*.

### What this study adds

- *Hypertension and diabetes were the most common co-morbidities in hospitalized COVID-patients;*
- *Case fatality rate was high (27.1%);*
- *Factors independently associated with mortality were heart failure, low systolic blood pressure and elevated blood glucose*.
